# Technological variables predictors of academic stress in nursing students in times of COVID-19

**DOI:** 10.1590/1518-8345.6386.3852

**Published:** 2023-05-12

**Authors:** Jhon Alex Zeladita-Huaman, Sonia Celedonia Huyhua-Gutierrez, Henry Castillo-Parra, Roberto Zegarra-Chapoñan, Sonia Tejada-Muñoz, Rosa Jeuna Díaz-Manchay

**Affiliations:** 1 Universidad Nacional Mayor de San Marcos, Departamento Académico de Enfermería, Lima, Lima, Perú.; 2 Universidad Nacional Toribio Rodríguez de Mendoza de Amazonas, Facultad de Ciencia de la Salud, Amazonas, Amazonas, Perú.; 3 Universidad de San Buenaventura, Facultad de Psicología, Medellín, Antioquia, Colombia.; 4 Universidad María Auxiliadora, Escuela Profesional de Enfermería, Lima, Lima, Perú.; 5 Ministerio de Salud, Escuela Nacional de Salud Pública, Lima, Lima, Perú.; 6 Universidad Católica Santo Toribio de Mogrovejo, Facultad de Medicina, Lambayeque, Lambayeque, Perú.

**Keywords:** Psychological Stress, Computers, Age Groups, Nursing Students, Distance Education, Coronavirus Infections, Estrés Psicológico, Computadores, Grupos de Edad, Estudiantes de Enfermería, Educación a Distancia, Infecciones por Coronavirus, Estresse Psicológico, Computadores, Grupos Etários, Estudantes de Enfermagem, Educação à Distância, Infecções por Coronavírus

## Abstract

**Objective:**

to analyze which technological variables, derived from the use of electronic devices, predict academic stress and its dimensions in Nursing students.

**Method:**

analytical cross-sectional study carried out with a total of 796 students from six universities in Peru. The SISCO scale was used and four logistic regression models were estimated for the analysis, with selection of variables in stages.

**Results:**

among the participants, 87.6% had a high level of academic stress; time using the electronic device, screen brightness, age and sex were associated with academic stress and its three dimensions; the position of using the electronic device was associated with the total scale and the stressors and reactions dimensions. Finally, the distance between the face and the electronic device was associated with the total scale and size of reactions.

**Conclusion:**

technological variables and sociodemographic characteristics predict academic stress in nursing students. It is suggested to optimize the time of use of computers, regulate the brightness of the screen, avoid sitting in inappropriate positions and pay attention to the distance, in order to reduce academic stress during distance learning.

## Abstract (es)

**Objetivo:**

analizar cuáles son las variables tecnológicas, derivadas del uso de dispositivo electrónico, predicen el estrés académico, y sus dimensiones en estudiantes de enfermería.

**Método:**

estudio transversal de tipo analítico, realizado en 796 estudiantes de seis universidades de Perú. Se empleó la escala SISCO y para el análisis se estimaron cuatro modelos de regresión logística, con selección de variables por pasos.

**Resultados:**

entre los participantes, 87,6% presentaron un nivel alto de estrés académico; el tiempo de uso del dispositivo electrónico, el brillo de la pantalla, la edad y el sexo, estuvieron asociados con el estrés académico y sus tres dimensiones; la posición de uso del dispositivo electrónico estuvo asociada con la escala total y con las dimensiones estresores y reacciones. Finalmente, la distancia entre el rostro y el dispositivo electrónico estuvo asociada con la escala total y la dimensión reacciones.

**Conclusión:**

las variables tecnológicas y las características sociodemográficas predicen el estrés académico en estudiantes de enfermería. Se sugiere optimizar el tiempo de uso de las computadoras, regular el brillo de la pantalla, evitar sentarse en posiciones inadecuadas y vigilar la distancia, con la finalidad de reducir el estrés académico durante la enseñanza a distancia.

## Abstract (pt)

**Objetivo:**

analisar quais variáveis tecnológicas, derivadas do uso de dispositivos eletrônicos, predizem o estresse acadêmico e suas dimensões em estudantes de enfermagem.

**Método:**

estudo transversal do tipo analítico, realizado em 796 estudantes de seis universidades do Peru. Foi utilizada a escala SISCO e foram estimados quatro modelos de regressão logística para a análise, com seleção das variáveis por etapas.

**Resultados:**

entre os participantes, 87,6% apresentaram alto nível de estresse acadêmico; o tempo de uso do aparelho eletrônico, o brilho da tela, a idade e o sexo foram associados ao estresse acadêmico e suas três dimensões; a posição de uso do aparelho eletrônico foi associada à escala total e às dimensões estressores e reações. Por fim, a distância entre o rosto e o dispositivo eletrônico foi associada à escala total e à dimensão das reações.

**Conclusão:**

variáveis tecnológicas e características sociodemográficas predizem estresse acadêmico em estudantes de Enfermagem. Sugere-se otimizar o tempo de uso dos computadores, regular o brilho da tela, evitar sentar-se em posições inadequadas e atentar-se à distância da tela, a fim de diminuir o estresse acadêmico durante o ensino a distância.

**Highlights:**

**(1)** Nursing students have a high level of academic stress.

**(2)** Computer usage time is a predictor of academic stress.

**(3)** Computer screen brightness is a predictor of academic stress.

**(4)** Being between 30 and 39 years old and being a man is a protective factor against academic stress.

**(5)** Study carried out in six Peruvian universities.

## Introduction

All over the world, the COVID-19 pandemic had a negative effect on the education of Health Sciences students, due to several factors. One of these aspects was the widespread use of new information technologies, since face-to-face classes were suspended and distance learning was implemented ^( 1)^ . Likewise, a study that evaluated academic stressors - during the first week of transition to online education - reported that access to the internet and difficulties in using digital platforms was the second most frequent factor and the third one for women ^( 2)^ . 

The interrelationships between people and machines, described as Human Computer Interface (HCI) and studies of cognitive ergonomics and usability, explain how certain aspects of interface design help or hinder this interaction. This causes stress, which facilitates or hinders learning and influences people’s motivation in areas such as teaching, work and social interactions ^( 3)^ . From an adaptive point of view, stress is an endocrine, immunological and behavioral response to the presence of an environmental stimulus or threat ^( 4)^ . This psychological construct, in the educational context, has been described as academic stress (AS), which is approached as a systemic process in three stages: perception of stressful stimuli, symptoms that indicate systemic imbalance and strategies that the student uses to face stress and restore the systemic balance ^( 5)^ . 

Studies prior to the pandemic, carried out with university students, indicate satisfaction with life, locus of control and gender as predictors of AS ^( 6)^ ; and, as a protection factor, the practice of physical activity ^( 7)^ . While studies carried out during the transition to distance learning, considering the changes in the teaching modality, motivated by COVID-19, increased the level of AS ^( 2)^ ; on the other hand, students who had access to a support program – offering the necessary resources for distance learning – had lower levels of AS ^( 8)^ . In addition, it has been reported that stress is associated with family income, having a private space to study and the type of electronic device used ^( 9)^ . However, there are few studies that evaluate the effect of electronic device (ED) use conditions on AS. 

For this reason, identifying the aspects that cause stress in computer-using students, although technically challenging, is extremely important in the educational context, especially in the distance learning scenario, where the use of EDs has become widespread. Based on this premise, the following research question was raised: what is the relationship between the conditions of use of EDs and AS in nursing students? Thus, this study aimed to analyze which technological variables, derived from the use of EDs, predict academic stress and its dimensions in nursing students.

## Method

### Study design

This is a quantitative and cross-sectional study, of analytical type, guided by the Standards for Quality Improvement Reporting Excellence (SQUIRE) tool.

### Research setting

The study was carried out in the city of Lima, Peru. Nursing students from six universities (3 public and 3 private) located in five cities in Peru (Lima, Amazonas, Ayacucho, Chiclayo and Cajamarca) were invited to participate. The study took place from May to July 2021.

### Population and sample

The population consisted of 1945 students. Inclusion criteria were being enrolled between the first and tenth semester of the Nursing course at the universities participating in the study, and having ED with internet access. Those under 18 years old were excluded. The calculated sample consisted of 796 students – selected by non-probabilistic sampling.

### Instrument

The survey technique was used through a virtual questionnaire. In the first section of the instrument, the informed consent form was requested, which included a question asking about the desire to participate in the study.

In the study, the conditions of use of the ED used to attend classes (time using it, distance between the face and the ED, position when using the ED, screen brightness, rest duration and use of the screen filter) were considered as possible predictors of AS, as well as sociodemographic variables (age, sex and occupation), use of glasses, knowledge and practice of the 20-20-20 rule of ophthalmology.

The AS level was determined using the SISCO ^( 4)^ inventory, consisting of 21 items, grouped into three dimensions: stressors, symptoms and coping strategies (7 items for each dimension). Their response options ranged from never = 0 points to always = 5 points. A low AS level (from 0 to 71 points) and a high AS level (from 72 to 105 points) were considered. The original scale was validated by 20 experts. Generic validity reports Aiken’s V concordance coefficients greater than 0.75; construct validity reports corrected Pearson’s r correlation coefficients greater than 0.2; and, a Cronbach’s Alpha of 0.85. Subsequently, the scale was validated in Peruvian ^( 10)^ and Chilean ^( 5)^ university students. In addition, with the collected data, it was determined that the scale has adequate reliability with a Cronbach’s alpha of 0.885. 

### Data collection procedures

Data collection was carried out using a form prepared in Google Forms, published on the social networks of health institutions, scientific nursing societies and universities that offer graduate programs in Nursing, between April and June 2021. The approximate time to complete the form was 30 minutes.

### Data analysis

Initially, descriptive analyzes of the study variables were performed. Subsequently, the AS scale and its dimensions were dichotomized, that is, people with low and no levels of stress were categorized as having low stress, and people with moderate and severe levels were categorized as having high stress. After that, 4 Logistic Regression models were performed to predict AS and its dimensions in nursing students. The predictor variables were chosen using a stepwise selection algorithm, which determines, through changes in the Akaike Information Indicator (AIC), the variables that have the best predictive capacity on the response variable. In the resulting models, a diagnosis of outliers and assumptions was made, and it was observed that no case was atypical and all the assumptions of the logistic regression were met. To interpret the logistic regression parameters, it was necessary to exponentiate the coefficients and read them in terms of odds ratio. All analyzes were performed using the R v4.0.1 ^( 11)^ software. 

### Ethical aspects

The research was approved by the Research Ethics Committee of the *Universidad Nacional Toribio Rodríguez de Mendoza de Amazonas* (letter n 001-2021). The ethical principles of dignity, autonomy and free will to participate in the study were respected, upon approval of the Informed Consent Form, in line with the ethical principles required by Peruvian regulations. 

## Results

The questionnaire was answered by a total of 796 Nursing students, among which 80.5% (641) were women. Likewise, most were aged between 20 and 29 years old (67.8%) and dedicated themselves only to studies (55.0%).

The ED most used by nursing students during their online classes was the computer (78.7%). Regarding the time of use of the ED, most participants used it for more than 4 hours a day (84.3%). In general, participants use their ED at a distance of 30 to 50 cm (55.4%). Regarding the position they adopt when using the ED, two thirds of the participants reported sitting and leaning in front of their ED (64.6%). The description of the other variables considered in the study is presented in Table 1. 

**Table 1 -:** Frequencies and percentages of variables related to the use of electronic devices by nursing students (n=796). Lima, Peru, 2021

Variables	n	%
Main ED* used for the classes		
Computer	627	78.7
Tablet	10	1.3
Smartphone	159	20.0
Time using the ED* during classes (daily hours)		
Between 1 and 4	125	15.7
More than 4	671	84.3
Distance between face and ED* (cm)		
Less than 30	315	39.6
30-50	441	55.4
More than 50	40	5.0
Position when using the ED*		
Sitting leaning	514	64.6
Sitting upright	262	32.9
Lying down	20	2.5
Screen brightness		
Opaque	177	22.2
Brilliant	520	65.3
Very bright	99	12.5
Use of glasses when using the ED*		
Yes	385	48.4
No	411	51.6
Rest duration (minutes)		
1 to 5	240	30.1
6 to 10	287	36.1
11 to 19	198	24.9
More than 20	71	8.9
Use of screen filter		
No	593	74.5
Sometimes	121	15.2
Yes	82	10.3
Knowledge of the 20-20-20 rule		
Yes	105	13.2
No	691	86.8
Application of the 20-20-20 rule		
Yes	50	6.3
No	746	93.7

* ED = Electronic device

The highest percentage of nursing students (86.6%) had a high level on the total AS scale and only 13.4% had a low level. A similar result was found in dimensions. On the scale of stressors, 80.2% reported a high level and 19.8% a low level; on the reaction scale, 73.7% had a high level and 26.3% had a low level. Finally, on the confrontation scale, 83.7% had a high level and 16.3%, a low level.

Table 2 presents the logistic regression model resulting from the selection of variables in stages for predicting the total AS scale. For this model, the algorithm selected the variables sex, age, time using it, distance from the face, position when using it and screen brightness. It is observed that people who use the ED for more than 5 hours a day have a 2.85 (p < 0.001) times greater odds ratio of having high AS than those who use the ED for less time. On the other hand, people who use EDs sitting upright have a 58% (p < 0.001) lower odds ratio of having high levels of stress than people who use their devices while sitting at an angle. People between 30 and 39 years old have a 62% (p < 0.01) lower odds ratio of having elevated AS than people between 18 and 20 years old. Likewise, people over 40 years old have a 67% (p < 0.05) lower odds ratio of having high levels of AS than people between 18 and 20 years old. Likewise, people who watch the ED screen in very bright mode have a 5.66 (p < 0.01) higher odds ratio of having high levels of AS than people who use it in dark mode. Regarding gender, men have a 53% (p < 0.01) lower odds ratio of having high levels of stress than women. Finally, people who use their EDs at a distance of 30-50 cm from their face have a 1.85 (p < 0.05) times greater odds ratio of having high levels of AS than people who use EDs at a shorter distance. 

**Table 2 -:** Logistic regression with stepwise variable selection for predicting the total scale of academic stress in nursing students (n=796). Lima, Peru, 2021

Variable	b*	*SE †*	*Exp (b) ‡*
Intercept	1.02 ||	0.43	2.78
Time of use of the ED § (>5 hours)	1.04 ¶	0.26	2.85
Position when using ED § (sitting upright)	-0.87 ¶	0.23	0.42
Position when using the ED § (lying down)	-0.83	0.63	0.44
Age (20-29 years old)	0.10	0.30	1.11
Age (30-39 years old)	-0.96 ||	0.38	0.38
Age (>40 years old)	-1.12	0.63	0.33
Screen brightness (bright)	0.39	0.25	1.49
Screen brightness (very bright)	1.73**	0.64	5.66
Sex (Male)	-0.76**	0.27	0.47
Distance between the face and DE § (30-50 centimeters)	0.61 ||	0.24	1.85
Distance between face and DE § (>50 centimeters)	0.27	0.47	1.31

* b = Non-standard coefficient;

† SE = Standard error;

‡ B = Standard coefficient;

§ ED = Electronic device;

|| P value < 0.05;

¶ P value < 0.001;

** P value < 0.01

Table 3 presents the logistic model resulting from the stepwise selection algorithm for predicting the stressor dimension. For this model, the variables age, gender, distance from the face, resting time, position of use and time of use of the ED were selected. Regarding the position, people who use the ED sitting upright have a 64% (p < 0.001) lower odds ratio of having a high level of stressors compared to those who use the ED sitting inclined. Furthermore, students who use the devices for more than 5 hours have a 2.53 (p < 0.01) higher odds ratio of reporting high levels of stressors than those who use the device for less than 5 hours. People between 30 and 39 years old have 53% (p < 0.05) and those over 40 years old have 85% (p < 0.001) lower odds ratio of having a high level of stressors than people between 18 and 20 years old. Men have a 49% (p < 0.01) lower odds ratio of having a high level of stressors than women. Regarding brightness, people who use the ED in very light mode have a 4.32 (p < 0.01) higher odds ratio of having a high level of stressors than those who use the ED in the opaque mode. Finally, students who take breaks between 10 and 19 minutes have an odds ratio 1.77 (p < 0.05) times greater for having a high level of stressors than those who take breaks of less than 6 minutes. However, people who take breaks of more than 20 minutes have a 49% (p < 0.05) lower odds ratio of having high levels of stress compared to those who take breaks of less than 6 minutes. 

**Table 3 -:** Logistic regression with selection of stepwise variables for predicting the stressors dimension in nursing students (n=796). Lima, Peru, 2021

Variable	b*	*SE †*	*Exp (b) ‡*
Intercept	0.76	0.41	2.15
Position when using the ED § (sitting upright)	-1.02||	0.20	0.36
Position when using the ED § (lying down)	-0.73	0.56	0.48
Time using the ED § (>5 hours)	0.92 ||	0.24	2.53
Age (20-29 years old)	0.13	0.25	1.14
Age (30-39 years old)	-0.75 ¶	0.35	0.47
Age (>40 years old)	-1.89**	0.61	0.15
Sex (Male)	-0.67**	0.24	0.51
Screen brightness (bright)	0.15	0.22	1.17
Screen brightness (very bright)	1.46**	0.49	4.32
Rest duration (6-10 min)	0.19	0.24	1.22
Rest duration (11-19 min)	0.57 ¶	0.28	1.77
Rest duration (>20 min)	-0.68 ¶	0.34	0.51
Distance between face and ED § (30-50 cm)	0.40	0.21	1.50
Distance between face and ED § (>50 cm)	-0.19	0.41	0.83

* b = Non-standard coefficient;

† SE = Standard error;

‡ B = Standard coefficient;

§ ED = Electronic device;

|| P value < 0.001;

¶ P value < 0.05;

** P value < 0.01

Table 4 shows the model resulting from the logistic regression analysis with stepwise selection to predict the AS reaction dimension. The algorithm selected for this model the variables age, sex, occupation, screen brightness, position when using and time using the ED. People who use the EDs in an upright sitting position were found to have a 59% (p < 0.001) lower odds ratio of reporting a high level of AS reactions than people who use the ED in a leaning position. Regarding age, those between 30 and 39 years old had a 47% (p < 0.05) lower odds ratio of reporting a high level of AS reactions than those between 18 and 20 years old. On the other hand, people who use the ED for more than 5 hours a day reported a 2.08 (p < 0.01) higher odds ratio of developing a high level of AS reactions than those who use their devices for less than 5 hours. People using the ED in very light mode are 3.91 (p < 0.001) times more likely to have a high level of AS reactions than those using the ED in dark mode. Men have a 48% (p < 0.01) lower odds ratio of having a high level of AS reactions. 

**Table 4 -:** Logistic regression with selection of stepwise variables to predict the reaction dimension in nursing students (n=796). Lima, Peru, 2021

Variable	b*	*SE †*	*Exp (b) ‡*
Intercept	0.73 ||	0.41	2.09
Position when using ED § (sitting upright)	-0.89 ¶	0.20	0.41
Position when using ED § (lying down)	-0.36	0.56	0.69
Age (20-29 years old)	0.30	0.24	1.36
Age (30-39 years old)	-0.63	0.25	0.53
Age (>40 years old)	-1.00	0.35	0.37
Time using the ED § (>5 hours)	0.73**	0.61	2.08
Screen brightness (bright)	0.23	0.24	1.27
Screen brightness (very bright)	1.36 ¶	0.22	3.91
Sex (Male)	-0.65**	0.49	0.52
Occupation (study and work)	-0.37	0.24	0.69

* b = Non-standard coefficient;

† SE = Standard error;

‡ B = Standard coefficient;

§ ED = Electronic device;

|| P value < 0.05;

¶ P value < 0.001;

** P value < 0.01

Finally, Table 5 presents the final logistic regression model with selection of stepwise variables for predicting the AS coping dimension. The algorithm selected for this model the variables gender, age, screen brightness, time using it and distance between the face and the ED. Students who use the ED in light mode have an odds ratio 1.78 (p < 0.01) times greater to present a high level of AS coping than people who use the device in opaque mode. Likewise, people who use the ED screen in very bright mode have a 4.50 (p < 0.01) times greater odds ratio of presenting a high level of AS coping than those who use the cellphone in opaque mode. On the other hand, people who use their devices for more than 5 hours have an odds ratio 1.77 (p < 0.05) times greater to present a high level of AS coping than those who use their devices for less time. Regarding gender, men have a 50% (p < 0.01) lower odds ratio of having a high level of coping with AS than women. People who hold their devices at a distance of 30 to 50 centimeters have a 1.68 (p < 0.05) higher odds ratio of having a high level of AS coping than those who hold their devices at shorter distances. Finally, people between 30 and 39 years old have a 56% (p < 0.05) lower odds ratio of having a high level of coping with AS than those between 18 and 20 years old. 

**Table 5 -:** Logistic regression with selection of stepwise variables to predict the dimension of coping among nursing students (n=796). Lima, Peru, 2021

Variable	b*	*SE †*	*Exp (b) ‡*
Intercept	0.67	0.41	1.96
Screen brightness (bright)	0.57 ||	0.20	1.78
Screen brightness (very bright)	1.50 ||	0.56	4.50
Time using the DE § (>5 hours)	0.56 ¶	0.24	1.77
Gender (Male)	-0.70 ||	0.25	0.50
Distance between face and ED § (30-50 cm)	0.51 ¶	0.35	1.68
Distance between face and ED § (>50 cm)	0.20	0.61	1.23
Age (20-29 years old)	-0.02	0.24	0.99
Age (30-39 years old)	-0.81 ¶	0.22	0.44
Age (>40 years old)	-0.42	0.49	0.66

* b = Non-standard coefficient;

† SE = Standard error;

‡ B = Standard coefficient;

§ ED = Electronic device;

|| P value < 0.001;

¶ P value < 0.05

## Discussion

In this study carried out with Nursing students who were in distance learning classes, in times of a pandemic due to the COVID-19, the main finding was that the position of the student during the use of the ED, the time using the ED, the distance and brightness of the ED screen were predictors of the AS level and its dimensions. In the field of cognitive ergonomics, this finding highlights the influence that the conditions of use of electronic devices exert on AS ^( 3)^ . Likewise, a systematic review reported an association between the use of Information and Communication Technologies (ICT) with technostress in different study designs in people who work with computers ^( 12)^ . 

The findings of this study demonstrate the need for universities, through different departments or areas, such as university assistance and/or tutoring, to implement strategies that help students to reduce AS or other mental health problems. The objective is to promote learning in times of a pandemic by COVID-19. Likewise, make teachers aware so that, when planning their distance learning sessions, they avoid scheduling prolonged time in front of the screen, being able to schedule active breaks.

Among the conditions of using the device, the greatest risk factor was the brightness of the ED screen, both in relation to the AS level and in all dimensions. This can occur because a computer emits high-energy electromagnetic radiation or blue light, which can stress the ciliary muscle in the eye. Eventually, continuous exposure to the computer screen can cause ocular stress ^( 13)^ . 

As no previous studies were found in students that evaluated this association, this finding may be in agreement with an investigation carried out in simulated scenarios, in which exposure to natural light (medium brightness) of a virtual forest could significantly reduce the participants’ stress, compared to very light levels ^( 14)^ . Likewise, an experiment that sought to reduce stress through devices that displayed empathy - to reduce the effect of negative stress, through the use of blue light - showed that the simple addition of blue light tends to reduce mental stress ^( 3)^ . This compared to the normal state can be interpreted, concluding that, in humans, the experiment without light induced more stress than the experiment with blue light. It is deduced that blue light can help maintain a lower level of stress. 

However, the association between screen brightness and stress reported in this study should be viewed with caution, as brightness was determined by the participant based on their perception, without any reference parameter. Thus, it is recommended that, for future studies that evaluate this variable, uniform criteria should be considered, such as consulting the possibility of dividing the ED brightness bar into parts.

Another factor that increased the risk of AS - both in the total scale and in all dimensions, reported in this study - was the time using the ED. Likewise, a study carried out in China reports that female students, who spend more than six hours a day in front of the computer, have a higher level of stress, but this association was not observed in men ^( 15)^ . Likewise, another study carried out in 38 countries in Europe and North America reports that adolescents who use computers to play games for more than 4 hours are at greater risk of stress ^( 16)^ . Among the possible reasons that explain this association, it can be considered that if the student, during online education, spends more time in front of an ED, this may increase their sedentary lifestyle and have less time to rest or perform other recreational activities ^( 15)^ . 

In line with previous studies, it was found that students who adopt the upright sitting posture, when using the ED, have a lower level of AS and levels of stressors and reactions. This finding is explained by the fact that sitting in this position improves blood circulation in the body and reduces the distance between the eye and the computer, which minimizes the electromagnetic radiation emitted by the computer ^( 13)^ . 

Regarding sociodemographic characteristics, in this study, it was found that men are less likely to have AS than women. This result coincides with studies prior to the COVID-19 pandemic ^( 6- 7)^ and with a recent study, which indicated that women have a higher level of AS, in all factors evaluated. Among them, the factor related to the methodological difficulties of the teacher and the overload of the student ^( 17)^ stands out. This shows that sociodemographic characteristics such as age and gender are mediating factors in the association between technological factors and AS. 

Likewise, it was found in the study that students aged between 30 and 39 years old have a lower risk of presenting a high level of AS and in all assessed dimensions, compared to students aged between 20 and 29 years old. This effect is increased among students over 40 years old, both in terms of AS and in terms of stressors. However, although this finding confirms the association between age and stress, additional studies are needed, as a systematic review showed that there is no linear trend between age and technostress ^( 12)^ . Likewise, another study verified that there is no association between the study year and the AS ^( 17)^ . 

According to previous research, university students showed a high level of academic stress in times of the COVID-19 pandemic ^( 2, 8)^ . Considering that, in times of a pandemic, university students spend many hours on their cell phones, laptops and other types of equipment, it is important to promote technology design so that it is as relaxing and pleasant as possible. The electronic instrument must have empathic capacity and meet the needs of its users. Screen-mediated education, pressure at work, social pressure, and the fast-paced world in general can cause stress. However, it is important to note that there are two types of stress, positive stress called eustress and negative stress called distress. These are well known terms to professionals who deal with mental health. 

Eustress can motivate and help humans to be more productive. When the demands placed on an individual (physical or psychological) are too high, performance begins to decline and the individual begins to experience negative stress, that is, distress. Anxiety can make people feel sick ^( 18)^ , greatly reducing productivity or even causing depression and exhaustion ^( 19)^ . There are different ways to make electronic equipment stimulate empathy. One example is the empathetic partners, who are virtual robots with human-like capabilities ^( 20)^ . Others may be empathic chatbots, which generate empathic responses used to diagnose and treat mental illnesses ^( 21)^ . Other ways that can be used are light and color, which can impact the emotional state of human beings ^( 22)^ . The intention is to create a system of interfaces applied to education and learning, to reduce stress by understanding how the student feels by generating empathic responses. 

This study had some methodological limitations that refer to the use of glasses, it was not asked if it was prescribed by an ophthalmologist or if it was self- medication. In addition, a scale was not used to determine the luminosity, only the perception that the students had was questioned. In addition, regarding the distance from the electronic device, it is necessary to differentiate the type of device. On the other hand, information was collected through self-report, which introduces a bias in the analysis. However, the strengths of the study were the use of a validated questionnaire and the survey of students from six universities, located in different areas of Peru.

This study is relevant and important to understand how it affects the brain: stress caused by new technologies, changes in technology-mediated education, and, the ruptures of current society. First, we know that technological progress advances faster than the plastic development of the human brain to adapt to changes. Second, because we are just beginning the development phase of the BCI (Brain Computer Interface) that will be mediated by: artificial intelligence, virtual reality, big data, the internet of things and the metaverse, among others. All these changes, although possibly positive for humanity, will affect the brain, putting strong pressure on it to adapt quickly. It is obvious that these modifications will involve effort, stress, cognitive flexibility, creation of executive functions and high social cognition. Studying how our mind (a set of conscious and unconscious activities and psychic processes, mainly of a cognitive nature) and our neural networks will be is a very important factor to prepare us for the near future that will decisively influence our behavior.

## Conclusion

Nursing students from Peru who were receiving distance learning, implemented as a result of the COVID-19 pandemic, spending more time using the ED, using the screen in very bright mode, aged between 30 and 39 years old and male were investigated. The above characteristics indicated a high level of AS in its three dimensions. It was found that the upright sitting position adopted by the student, while using the ED, significantly reduces the risk of presenting a high level of AS, both in the total scale and in the dimensions of stressors and reactions. Likewise, using the ED at a distance of 30 to 50 centimeters in relation to the face increases the risk of presenting a high level of AS in the total scale and in the confrontation dimension. Finally, the duration of a break longer than 20 minutes reduces the risk of presenting a high level of AS only in the stressor dimension.

Based on the findings, we conclude that in order to reduce the high level of AS, presented by Nursing students who are in online learning, it is suggested that professors optimize the time of use of computers during learning sessions. Also, university authorities develop strategies that promote the regulation of screen brightness and, finally, preventing students from using EDs in inappropriate positions and keeping the proper distance.

Due to the COVID-19 pandemic, students are using ED more frequently to learn, work and socialize, for longer and longer periods of time. Therefore, it is suggested to continue investigating the impact of the conditions of use of EDs on people’s emotional state, at various levels of education.
